# T helper cell responses in adult diarrheal patients following natural infection with enterotoxigenic *Escherichia coli* are primarily of the Th17 type

**DOI:** 10.3389/fimmu.2023.1220130

**Published:** 2023-09-20

**Authors:** Marjahan Akhtar, Salima Raiyan Basher, Nuder Nower Nizam, Lazina Hossain, Taufiqur Rahman Bhuiyan, Firdausi Qadri, Anna Lundgren

**Affiliations:** ^1^ Infectious Diseases Divison, icddr, b (International Centre for Diarrhoeal Disease Research, Bangladesh), Dhaka, Bangladesh; ^2^ Department of Microbiology and Immunology, Institute of Biomedicine, University of Gothenburg, Gothenburg, Sweden; ^3^ Department of Clinical Immunology and Transfusion Medicine, Sahlgrenska University Hospital, Gothenburg, Sweden

**Keywords:** ETEC, T helper cell, Th17, Th1, IL-17A, IFN-γ

## Abstract

**Background:**

Infection with enterotoxigenic *Escherichia coli* (ETEC) gives rise to IgA antibodies against both the heat labile toxin (LT) and colonization factors (CFs), which are considered to synergistically protect against ETEC diarrhea. Since the development of ETEC-specific long lived plasma cells and memory B cells is likely to be dependent on T helper (Th) cells, we investigated if natural ETEC diarrhea elicits ETEC-specific Th cells and their relation to IgA responses.

**Methods:**

Th cell subsets were analyzed in adult Bangladeshi patients hospitalized due to ETEC diarrhea by flow cytometric analysis of peripheral blood mononuclear cells (PBMCs) isolated from blood collected day 2, 7, 30 and 90 after hospitalization as well as in healthy controls. The LT- and CF-specific Th responses were determined by analysis of IL-17A and IFN-γ in antigen stimulated PBMC cultures using ELISA. ETEC-specific IgA secreted by circulating antibody secreting cells (plasmablasts) were analyzed by using the antibodies in lymphocyte supernatants (ALS) ELISA-based method and plasma IgA was also measured by ELISA.

**Results:**

ETEC patients mounted significant ALS and plasma IgA responses against LTB and CFs on day 7 after hospitalization. ETEC patients had significantly elevated proportions of memory Th cells with a Th17 phenotype (CCR6+CXCR3-) in blood compared to controls, while frequencies of Th1 (CCR6-CXCR3+) or Th2 (CCR6-CXCR3-) cells were not increased. Antigen stimulation of PBMCs revealed IL-17A responses to LT, most clearly observed after stimulation with double mutant heat labile toxin (dmLT), but also with LT B subunit (LTB), and to CS6 in samples from patients with LT+ or CS6+ ETEC bacteria. Some individuals also mounted IFN-γ responses to dmLT and LTB. Levels of LTB specific IgA antibodies in ALS, but not plasma samples correlated with both IL-17A (r=0.5, p=0.02) and IFN-γ (r=0.6, p=0.01) responses to dmLT.

**Conclusions:**

Our results show that ETEC diarrhea induces T cell responses, which are predominantly of the Th17 type. The correlations between IL-17A and IFN-g and intestine-derived plasmablast responses support that Th responses may contribute to the development of protective IgA responses against ETEC infection. These observations provide important insights into T cell responses that need to be considered in the evaluation of advanced ETEC vaccine candidates.

## 1 Introduction

Enterotoxigenic *Escherichia coli* (ETEC) is one of the most common causative agents of acute watery diarrhea in children and adults in low- and middle-income countries and is a key public health problem that results in considerable child mortality and morbidity (1). ETEC enters the gut through contaminated foods or water and subsequently attaches and colonizes the epithelial cells using different appendages present on the bacterial surfaces known as colonization factors (CFs) (2). So far over 25 different CFs have been identified and among them, CFA/I, CS5, CS6 and CS3 are often the most common (3, 4). ETEC produces heat-labile enterotoxin (LT) and/or heat-stable enterotoxin (ST), which modulate ion channels and increase the loss of salt and water into the intestinal lumen and ultimately cause watery diarrhea (2, 5).

ETEC is a non-invasive bacterium and protective immunity towards ETEC is considered to be mainly mediated by mucosal secretory IgA (SIgA) antibodies (2, 6). At present, there is no licensed vaccine available against ETEC. However, different preclinical and clinical trials with candidate ETEC vaccines are ongoing, including the oral vaccine ETVAX developed and evaluated by our study group (6, 7). The ETVAX vaccine contains inactivated *E. coli* bacteria expressing common CFs plus a LT-toxoid and is currently the most advanced vaccine candidate, currently undergoing Phase II testing, and Phase III studies are planned to be started soon (6, 8). Vaccination against ETEC mainly targets antibody mediated immunity. ETEC infection and vaccination both induce increased levels of mucosal and plasma antibodies against the B subunit of LT (LTB) and CFs (2, 6). Responses include transient increases in ETEC specific antibody secreting cells (ASCs; i.e. plasmablasts) in peripheral blood after antigenic exposure and development of memory B cell responses (6, 9, 10). Similar to in many other enteric infections, antigen specific ASCs detected in blood during early ETEC infection are considered good proxy measurement of a mucosal B cell response (9, 11–13). ETEC specific ASCs in blood correlate with the number of ASCs detected in intestinal biopsy specimens one week later (11) and ASCs specific for ETEC CFs and toxoid can also be efficiently isolated from peripheral blood of ETEC infected patients 7 days after onset of symptoms using magnetic beads coated with antibodies directed against the mucosal homing receptor α4β7 integrin (9). Natural ETEC infection provides protection against subsequent infection with homologous ETEC strains and memory B cells are likely to contribute to this protection (10, 14, 15).

The CD4+ T helper (Th) cells are well known for their role in the development, affinity maturation and isotype switching of antibody responses as well as in the generation of memory B cells following infection. However, T cell-mediated immune responses to natural ETEC infection remains to be described. In this study, we analyzed Th cells with a focus on Th17 responses, because of their important role in induction of mucosal immunity and protection against other gastrointestinal infections, including *Vibrio cholerae*, *Shigella, Salmonella*, *Klebsiella pneumoniae and Helicobacter pylori* (16–19). The Th17 cells are proinflammatory cells, characterized by the expression of the CCR6 chemokine receptor, which play a critical role in host defense and clearance of both extracellular and intracellular bacterial infections and in maintaining the mucosal epithelial barrier (20). Th17 cells typically produce the cytokine IL-17A, which is critical for the regulation of both protective and pathogenic immune responses, and may also promote the upregulation of polymeric immunoglobulin receptor on the epithelial cells to facilitate IgA secretion in the gut lumen (21). IL-17A is also required for IgA and IgG1 antibody production, stimulates fibroblasts and epithelial cells to secrete proinflammatory mediators, including G-CSF, GM-CSF, and IL-8, which have roles in neutrophil recruitment, granulopoiesis, and inflammatory responses (20, 22).

Our previous studies of Th responses to ETVAX suggest that the vaccine induces ETEC specific Th17 and Th1 cells as well as activated T follicular helper (Tfh)-like cells with predominantly a Th17 phenotype in the circulation of healthy Swedish adults (23) and Lundgren A. et al., unpublished). The Tfh-like responses were found to correlate with ETEC specific antibody responses in the vaccinees (23). The ETVAX vaccine has been administered together with the mucosal adjuvant double mutant LT (dmLT) (7, 24–27), which is also known to predominantly enhance Th17 responses to several types of vaccines and vaccine antigens, both *in vitro* and *in vivo* (28–31). dmLT is homologous to ETEC LT, except for two mutations in the enzymatically active A subunit, which inhibit the toxicity but not the adjuvanticity of the molecule (24). However, to what extent the T cell responses induced by the ETVAX vaccine plus dmLT adjuvant resemble T cell responses induced by natural ETEC infection is unclear at present. Considering the ability of ETEC infection to induce protective immunity and the importance of Th cells for induction of long-lived protective IgA responses in general, Th cell responses to natural ETEC infection is thus an important target for investigation.

The overall aim of this study was to investigate Th responses to natural ETEC infection and their relation to IgA antibody responses in hospitalized adult diarrheal patients, both during acute infection and the convalescence period. This in-depth analysis of Th responses after natural infection will provide important T cell benchmarks for evaluating leading ETEC vaccine candidates, including ETVAX, in future clinical studies and field trials.

## 2 Methods and materials

### 2.1 Study design

Diarrheal patients hospitalized at the International Centre for Diarrheal Disease Research, Bangladesh (icddr,b) in Dhaka, Bangladesh were screened for ETEC, *Vibrio cholerae* O1, *Shigella*, *Salmonella* and enteric parasites on the first day of hospitalization. Adult male and female patients (n=30) with stool specimens positive for ETEC, as determined by the presence of LT and/or ST enterotoxin genes using polymerase chain reaction (PCR) (32), but negative for other enteric pathogens, were enrolled (**Table 1**). Patients had experienced diarrhea for 6-96 hours (median 24 hours) prior to hospitalization and most had severe dehydration (83%). Expression of ETEC-specific CFs by the isolated ETEC strains was determined by a dot blot assay (33). Among the enrolled patients, 77% were infected with ETEC strains expressing both LT and ST, 10% were LT only and 13% were ST only (**Table 1**). A majority of isolated ETEC strains expressed known CFs; CS5+CS6+ and CFA/I+ strains were most common (37% and 20%, respectively), whereas strains expressing other CFs were less frequent (20%). In 20% of the strains, no CF was identified. In addition, healthy adult participants (n=21) who had no history of gastrointestinal disorders, diarrhea, febrile illness or antibiotic use during two weeks before enrollment, based on the clinical assessment by study physicians, were recruited from a similar urban area in Dhaka (**Table 1**). Informed written consent was obtained from each participant. The study was approved by the research review and the ethical review committees of icddr,b.

**Table 1:** Demographics of study participants.

Parameters	ETEC infected patients (n=30)	Healthy individuals (n=21)
Age, median (range), years	32 (18-55)	28 (18-39)
Sex, n (%)		
Male	16 (53%)	9 (43%)
Female	14 (47%)	12 (57%)
Duration of diarrhea, median (range), hours		
Prior to hospitalization	24 (6-96)	n/a
Total duration	49 (6-123)	n/a
Dehydration status, n (%)		
Severe^a^	25 (83%)	n/a
Mild^b^	4 (13%)	n/a
None	1 (3%)	n/a
Toxin types of ETEC strains, n (%)		
LT+ST	23 (77%)	n/a
LT	3 (10%)	n/a
ST	4 (13%)	n/a
Colonization factors of ETEC strains, n (%)		
CFA/I	6 (20%)	n/a
CS5+CS6	11 (37%)	n/a
CS6+CS8	1 (3%)	n/a
Other CFs (CS1, CS3, CS4 CS14 and CS7)	6 (20%)	n/a
Negative	6 (20%)	n/a

a Severe dehydration was defined according to WHO criteria as patients having at least two of the following signs: lethargy/unconsciousness, sunken eyes, unable to drink or drink poorly, skin pinch goes back very slowly (≥2 seconds).

b Mild dehydration was defined according to WHO criteria as patients having at least two of the following signs: restlessness, irritability, sunken eyes, drinks eagerly, thirsty.

‘n/a’ means ‘not applicable’.

### 2.2 Blood collection and processing

Heparinized venous blood was collected from ETEC infected patients at the acute stage of illness (the second day after hospitalization, day 2), at early convalescence (day 7, range day 6-10), and late convalescence (30 ± 7 days and 90 ± 7 days after hospitalization). From healthy individuals, blood specimens were collected once after enrollment. Plasma and peripheral blood mononuclear cells (PBMCs) were separated by density-gradient centrifugation using Ficoll-Isopaque (Pharmacia, Sweden). Plasma specimens were stored at -20°C. From subsets of patients, CD4+ cells were depleted from PBMCs using magnetic beads (Dynabeads, Invitrogen, USA), according to the manufacturer´s instructions. The frequency of CD4+ T cells among lymphocytes after depletion was <3% (mean 1%), as determined by flow cytometric analysis.

### 2.3 Cell culture and stimulation

For analysis of T cell responses, PBMCs were cultured in DMEM F12 medium (Thermo Fisher Scientific, USA) supplemented with 50 mg/ml gentamicin (Sigma, USA) and 5% human AB+ serum (Sigma-Aldrich) at 37°C in a 5% CO_2_ incubator. Whole PBMCs and PBMCs depleted of CD4+ cells (10^5^ cells/well) were cultured in duplicate wells in U-bottomed 96-well plates. Cells were left untreated or stimulated with phytohaemagglutinin (PHA,1 µg/ml, Remel, USA), dmLT (10 µg/ml), LTB (10 µg/ml), LTE112K (10 µg/ml), LT (10 µg/ml), CFA/I (1 µg/ml), CS5 (5 µg/ml) or CS6 (1 µg/ml). After 5 days culture, supernatants were collected and stored at -80°C for cytokine analysis. The indicated stimuli concentrations were found to induce maximal cytokine responses in initial set-up experiments testing a range of different concentrations. Details regarding production of different CF antigens, LTB and dmLT have been previously described (27). LTE112K, LT and LTB used in control experiments were kindly provided by J. Clements, Tulane University, USA.

For analysis of ETEC-specific ASCs, the antibodies in lymphocytes secretions (ALS) assay was used. Several studies have shown that results from the ALS assay, in which the levels of antigen-specific antibodies spontaneously secreted by peripheral blood ASCs *in vitro* are measured by ELISA, closely correlate with results from the more traditional ELISPOT assay where the numbers of antigen-specific ASCs are determined by counting spots developed on a membrane (13, 34–36). In the ALS assay, PBMCs were cultured at 10^7^ cells/ml in RPMI complete medium (Invitrogen, USA) supplemented with 10% fetal bovine serum (FBS, Invitrogen), Na-pyruvate (1mM, Invitrogen), L-Glutamine (2mM, Invitrogen), 100 IU/ml Penicillin (Invitrogen) and 0.1mg/ml Streptomycin (Invitrogen). Cells were incubated in 96-well flat-bottomed tissue culture plates (2 x 10^6^ cells/well) for 48 hours at 37°C with 5% CO_2_. Supernatants were collected and stored at -80°C.

### 2.4 ELISA analysis of cytokines and antibodies

The concentrations of IL-17A and IFN-γ were determined using sandwich ELISA (eBioscience, USA) following the manufacturer’s instructions. IgA antibodies specific for LTB, CFA/I, CS5 and CS6 in ALS and plasma specimens were analyzed by ELISA. Briefly, as described previously (26), to measure LTB-specific IgA antibodies in ALS/plasma specimens, 96-well ELISA plates (Nunc, USA) were coated with GM1 ganglioside (0.3 nmol/mL) overnight at room temperature followed by recombinant LTB subunit (1 μg/mL). To determine CF specific IgA antibody responses, high binding 96-well ELISA plates (Greiner, Austria) were coated with CFA/I (0.5 µg/mL), CS3 (0.3 µg/mL), CS5 (0.5 µg/mL) or CS6 (0.5 µg/mL). Plasma/ALS specimen was added to each well; initially diluted 1:10 (plasma LTB and CFs), 1:4 (ALS LTB) or 1:2 (ALS CFs) in PBS with 0.1% bovide serum albumin (BSA) and 0.05% Tween and a serially 3-fold dilution (plasma LTB and CFs and ALS LTB) or 2-fold (ALS CFs) was performed. The presence of antigen-specific antibodies was detected using horseradish peroxidase (HRP)-conjugated anti-human IgA (Jackson ImmunoResearch; 1:1500 dilution in 0.1% BSA-PBS-Tween) and ortho phenylenediamine (Sigma-Aldrich) in 0.1 M sodium citrate buffer (pH 4.5) and 30% hydrogen peroxide (Merck, USA). For plasma, the reactions were stopped after 20 minutes and endpoint titers were determined as the reciprocal interpolated dilutions of the samples at 450 nm that were 0.4 above background. For ALS, the reactions were stopped after 20 minutes by adding 1 M H_2_SO_4_ and endpoint titers were determined as the reciprocal interpolated dilutions of the samples at 492 nm that were 0.2 above background. All plasma, ALS and T cell supernatant samples from one individual collected at different time points were analyzed on the same plate in each type of assay and reference samples were included in all analyses to verify consistency between different runs.

### 2.5 Flow cytometric analysis

Fresh PBMCs (10^6^) were stained with Aqua fluorescent live/dead (Fixable Dead Cells Stain Kit, Invitrogen) to exclude dead cells. To analyze the frequency of different Th cell subsets, PBMCs were stained with anti-CD3-PETR (clone S4.1, Invitrogen), anti-CD4-PerCP (SK3, Becton Dickinson, USA), anti-CD45RO-PECy7 (UCHL1, BD), anti-CXCR3-PE (1C6/CXCR3, BD) and anti-CCR6-BV421 (11A9, BD). To analyze the purity of CD4-depleted PBMCs, cells were stained with anti-CD3-PETR (clone S4.1, Invitrogen) and anti-CD4-PerCP (SK3, BD). After staining, cells were acquired (100,000 total events) using FACSAria™ Fusion (BD, USA). Results were analyzed using FlowJo software (version 10, FlowJo LLC, BD, USA). Among the lymphocytes, 99% cells were found to be alive as determined by live/dead staining. Different subsets of live T cells were analyzed following the gating strategy shown in **Supplementary Figure 1**.

### 2.6 Statistical analyses

Statistical analyses were performed using GraphPad Prism 6 (GraphPad Software, Inc., USA). The Kruskal Wallis test with Dunn’s multiple comparison post-test was used to compare immunological responses in healthy controls and individual sampling time points of ETEC patients. The Mann-Whitney test was used to compare maximum responses at any time point between ETEC patients and healthy individuals. The increases (fold-rises) in magnitudes of immune responses in patients were calculated either as the antibody or cytokine levels found in patient samples on day 2, 7, 30 or 90 after hospitalization divided by the corresponding mean+2SD values in samples from healthy controls or as the antibody or cytokine levels found in patients on day 7, 30 or 90 after hospitalization divided by the corresponding levels on day 2, as indicated for each analysis. A responder was defined as having ≥2-fold increase in the magnitude of response. The Fisher’s Exact test was used to compare the responder frequencies at different time points. Correlation analyses were performed using the Spearman test. *P* values <0.05 were considered significant.

## 3 Results

### 3.1 Intestine derived and plasma IgA responses

**Figure 1:**
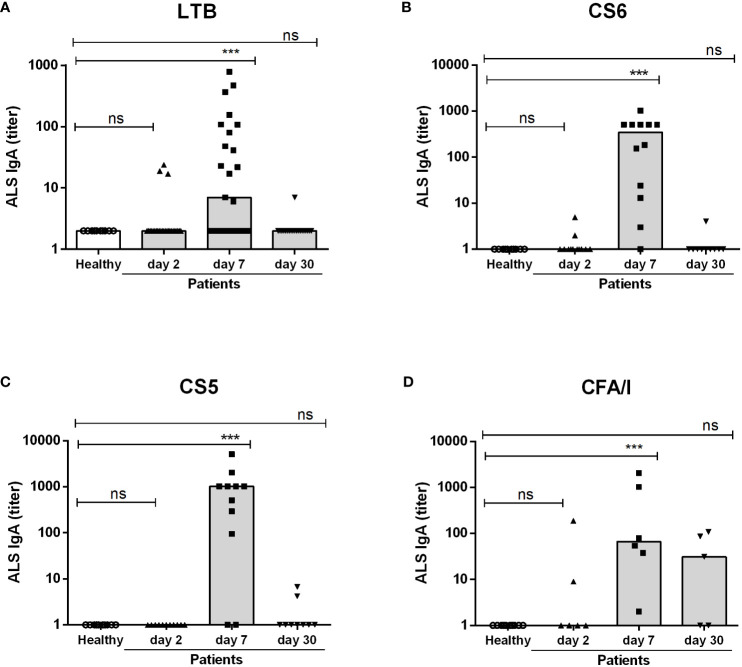
ALS IgA antibody responses to LTB and CFs in ETEC infected patients and healthy adults. **(A)** LTB-, **(B)** CS6-, **(C)** CS5- and **(D)** CFA/I-specific IgA titers were analyzed in ALS samples in healthy adults (n=10) and diarrheal patients on day 2, 7 and 30 after hospitalization **(A)**; n=20-26, **(B)**; n=10-12, **(C)**; 9-11 and **(D)**; n=5-6. Each symbol represents one individual, and bars indicate median values. Statistical analysis was performed using the Kruskal Wallis test with Dunn’s multiple comparison post test. ^***^ *P <*0.001, ns, not significant; *P >*0.05.

We first investigated the mucosal and plasma IgA responses to ETEC infection in adult hospitalized ETEC infected diarrheal patients. Mucosal responses were analyzed by measuring LTB- and CF-specific IgA antibodies secreted from intestine-derived ASCs migrating in peripheral circulation using the ALS method. Analysis of ASCs using ALS or ELISPOT has been shown to be a good correlate of mucosal B-cell responses in many studies of enteric infections and vaccines (11–13, 37). IgA antibodies specific for LTB and CFs most commonly expressed by the infecting strains (ETEC positive for CS6, CS5 and CFA/I) were analyzed in samples from both diarrheal patients infected with ETEC strains expressing the corresponding antigens and healthy controls. Two days after hospitalization, ALS IgA responses were low and infrequent in most patients, but 8-33% of the patients responded to LTB, CS6 and CFA/I with antibody levels at least 2-fold higher than those observed in healthy controls already at this early time point (**Figure 1** and **Table 2**). On day 7 after hospitalization, significant increases in levels of ALS IgA responses were observed against both LTB and all CFs compared to healthy individuals (**Figure 1** and **Table 2**). CF-specific ALS responses were more frequent (82-100%) than LTB responses (56%) at this time point (**Table 2**). Responses were low in most participants on day 30 except for IgA against CFA/I, which remained elevated in about half of the analyzed participants (P>0.05). The ALS antibody levels on day 7 and day 30 were also compared to levels measured in samples collected two days after hospitalization (corresponding to 6 hours to 4 days after onset of symptoms) (**Supplementary Table 1**). This control analysis gave similar responder frequencies as comparisons to the healthy controls for most antigens, but slightly lower responder frequencies to CFA/I.

**Table 2:** Frequencies of IgA antibody responders^a^ against LTB and CF antigens in ALS and plasma specimens from ETEC patients.

	Day 2	Day 7	P-value^b^	Day 30	P-value^b^	Day 90	P-value^b^	Cumulative (Day 2/7/30/90)
ALS								
**LTB**	3/26 (12%)	14/25 (56%)	0.001	1/20 (5%)	0.622	–	–	14/26 (54%)
**CS6**	1/12 (8%)	11/12 (92%)	<0.001	1/10 (10%)	1.000	–	–	11/12 (92%)
**CS5**	0/11 (0%)	9/11 (82%)	<0.001	2/9 (22%)	0.190	–	–	9/11 (82%)
**CFA/I**	2/6 (33%)	6/6 (100%)	0.061	3/5 (60%)	0.567	–	–	6/6 (100%)
Plasma								
**LTB**	4/26 (15%)	17/25 (68%)	<0.001	10/22 (45%)	0.029	4/19 (21%)	0.704	17/26 (65%)
**CS6**	0/12 (0%)	10/12 (83%)	<0.001	2/10 (20%)	0.195	0/8 (0%)	1.000	10/12 83%)
**CS5**	0/11 (0%)	8/11 (73%)	0.001	2/9 (22%)	0.190	0/7 (0%)	1.000	8/11 (73%)
**CFA/I**	1/6 (17%)	4/6 (67%)	0.242	2/5 (40%)	0.546	1/3 (33%)	1.000	4/6 (67%)

a A responder was defined as having ≥2-fold higher antibody titer compared to the mean+2SD titer of the group of healthy controls.

b Statistical analysis was performed using the Fisher’s Exact test to compare the responder frequency between day 7, 30 and 90 vs day 2.

**Figure 2:**
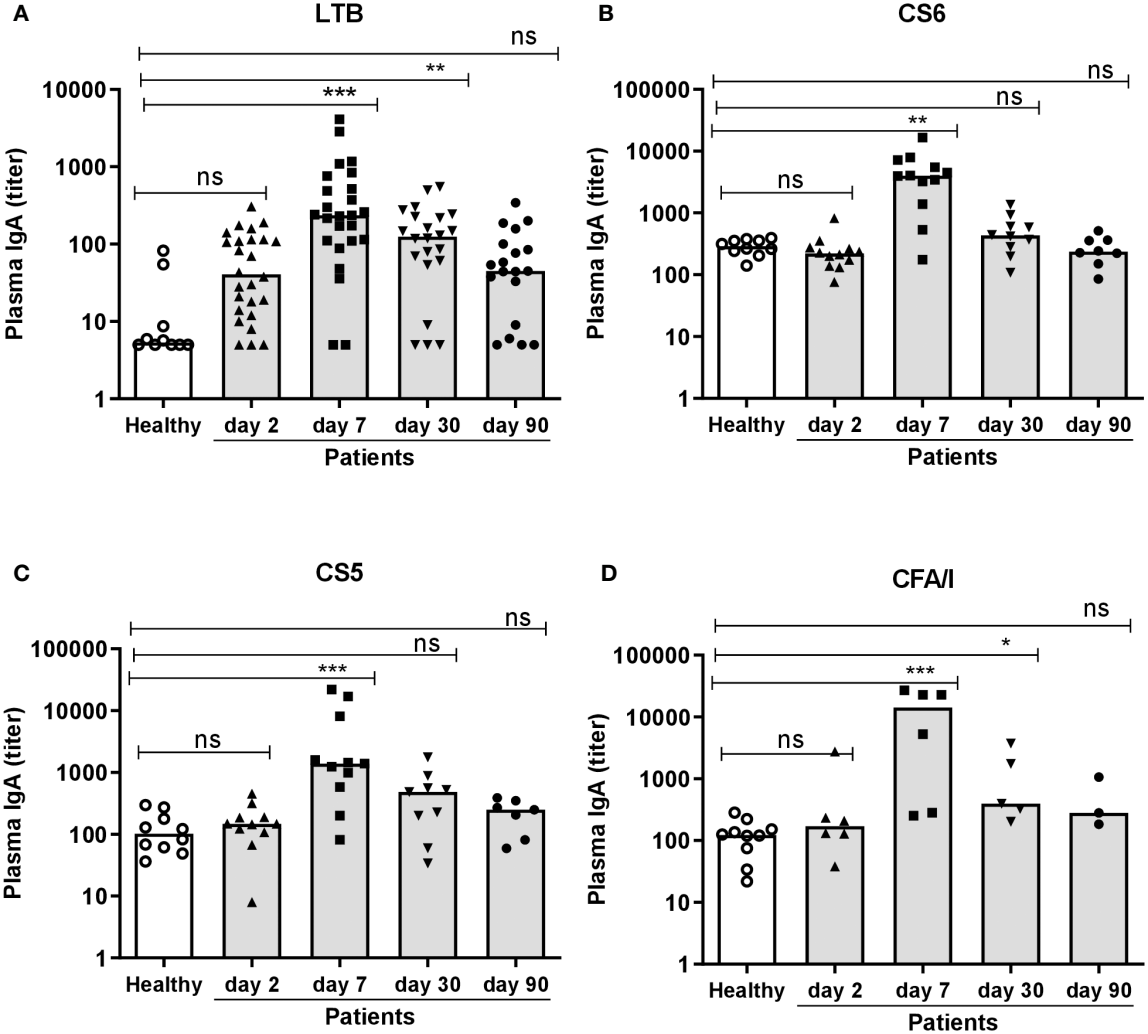
Plasma IgA antibody responses to LTB and CFs in ETEC infected patients and healthy adults. **(A)** LTB-, **(B)** CS6-, **(C)** CS5- and **(D)** CFA/I-specific IgA titers were analyzed in plasma samples in healthy adults (n=10) and diarrheal patients on day 2, 7, 30 and 90 after hospitalization **(A)**; n=19-26, **(B)**; n=8-12, **(C)**; 7-11 and **(D)**; n=3-6. Each symbol represents one individual, and bars indicate median values. Statistical analysis was performed using the Kruskal Wallis test with Dunn’s multiple comparison post test. ^*^ *P <*0.05, ^**^ *P <*0.01, ^***^ *P <*0.001, ns, not significant; *P >*0.05.

ETEC patients also mounted significant IgA responses in plasma. Magnitudes of IgA responses to LTB were increased already on study day 2 in 15% of the participants compared to healthy controls, whereas few patients responded to CFs in plasma at this early time point (**Figure 2** and **Table 2**). On day 7, magnitudes of responses to both LTB and all CFs were significantly higher in patients compared to healthy controls and reached the highest levels detected during the study period (**Figure 2**). Among the patients, 68% had at least 2-fold increased levels of LTB-specific IgA and 67-83% CF-specific IgA in plasma on day 7 compared to healthy controls (**Table 2**). The responder frequencies were slightly higher for most antigens when comparisons were made to levels measured on day 2 instead of to healthy controls (**Supplementary Table 1**). Plasma responses against all antigens declined until day 30, but LTB- (both magnitudes and responder frequencies) and CFA/I-specific (only magnitudes) responses still remained significantly elevated compared to levels in healthy controls at this time point (**Figure 2** and **Table 2**). All plasma responses declined to levels comparable to those observed in controls by day 90.

Collectively, these results show that a majority of the ETEC infected patients enrolled in the study mounted strong mucosal and plasma IgA responses to the LT and CFs expressed by the infecting ETEC strains, with maximal antibody levels detected on day 7 after hospitalization.

### 3.2 CD4+ memory Th cell responses

**Figure 3:**
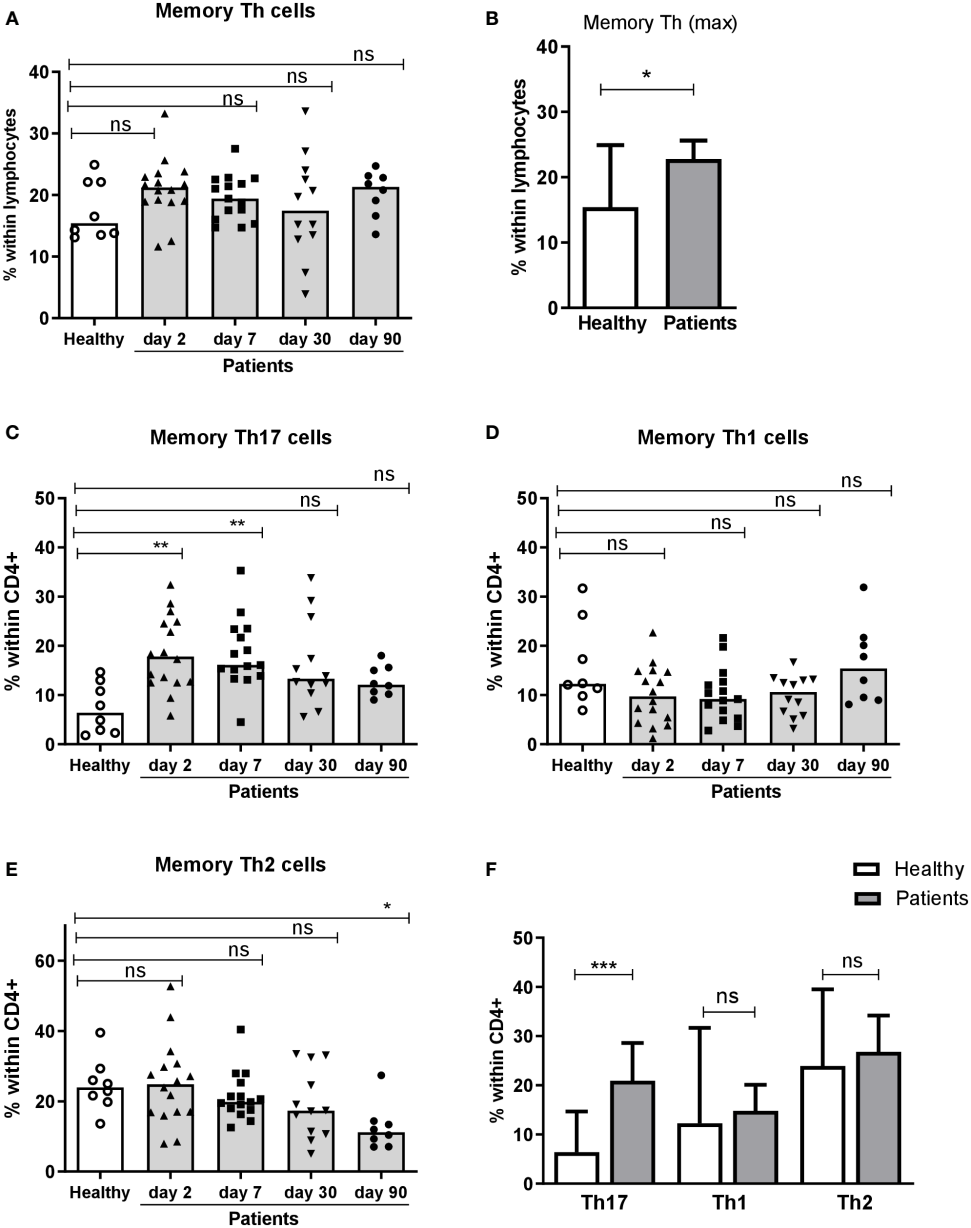
Frequencies of CD4+ memory Th cells and Th17, Th1 and Th2 subsets in peripheral blood of ETEC infected patients and healthy adults. Frequencies of **(A, B)** memory Th cells (CD4+CD45RO+) within lymphocytes, and **(C)** Th17 (CCR6+CXCR3-), **(D)** Th1 (CCR6-CXCR3+) and **(E)** Th2 (CCR6-CXCR3-) populations within CD4+ cells in PBMCs isolated from healthy adults (n=8) and ETEC infected patients on day 2, 7, 30 and 90 after hospitalization (n=8-16). **(A, C E)** Frequencies at each individual time point. Each symbol represents the percentage of cells for one individual, and bars indicate median values. **(B, F)** Maximum frequencies at any time point. Bars represent median + 95% confidence interval. Statistical analysis was performed by the Kruskal Wallis test with Dunn’s multiple comparison post test **(A, C, E)** or the Mann-Whitney test **(B, F)**. ^*^ *P <*0.05, ^**^ *P <*0.01, ^***^ *P <*0.001, ns, not significant; *P >*0.05.

To investigate the Th response to ETEC, we first analyzed the total frequencies of memory Th (CD4+CD45RO+) cells in fresh PBMCs collected from ETEC patients infected with ETEC strains expressing LT, CFA/I, CS5 or CS6 and healthy controls. Frequencies of memory Th cells among lymphocytes were comparable in ETEC patients and controls both in the acute and convalescence phase of infection (**Figure 3A**). However, when maximum frequencies of memory Th cells at any time point were compared with proportions in the healthy controls, a small but significant increase was observed in ETEC patients (**Figure 3B**), which was not seen when analyzing levels at each time point separately (**Figure 3A**).

Next, we analyzed the proportions of Th1, Th2 and Th17 subsets within memory Th cells based on the expression of the chemokine receptors CXCR3 and CCR6 in both patients and controls. The frequencies of Th17 (CCR6+CXCR3-) cells were significantly increased in patients already on day 2 compared to the healthy controls and Th17 responses remained significantly elevated until day 7 of infection (**Figure 3C**). The proportions of Th17 cells decreased on day 30-90. In contrast, ETEC diarrhea did not induce any increases in proportions of Th1 (CCR6-CXCR3+) or Th2 (CCR6-CXCR3-) cells at any time point (**Figures 3D, E**). Analysis of maximum frequencies of cells at any time point during infection gave the same result; strongly increased proportions of Th17 cells, whereas frequencies of the Th1 and Th2 subsets were comparable to the controls (**Figure 3F**). These flow cytometric results suggest that ETEC infection induces a Th response that is predominantly of the Th17 type, but did not reveal the antigen specificity of the responding cells.

### 3.3 LT-specific Th responses

To determine if the increased proportions of Th17 cells in the circulation of ETEC infected patients were due to LT- and/or CF-specific T cell responses, PBMCs from both patients and controls were stimulated with LTB, dmLT or CFs matching the infecting strain and the secretion of IL-17A and IFN-γ was analyzed in the culture medium.

**Figure 4:**
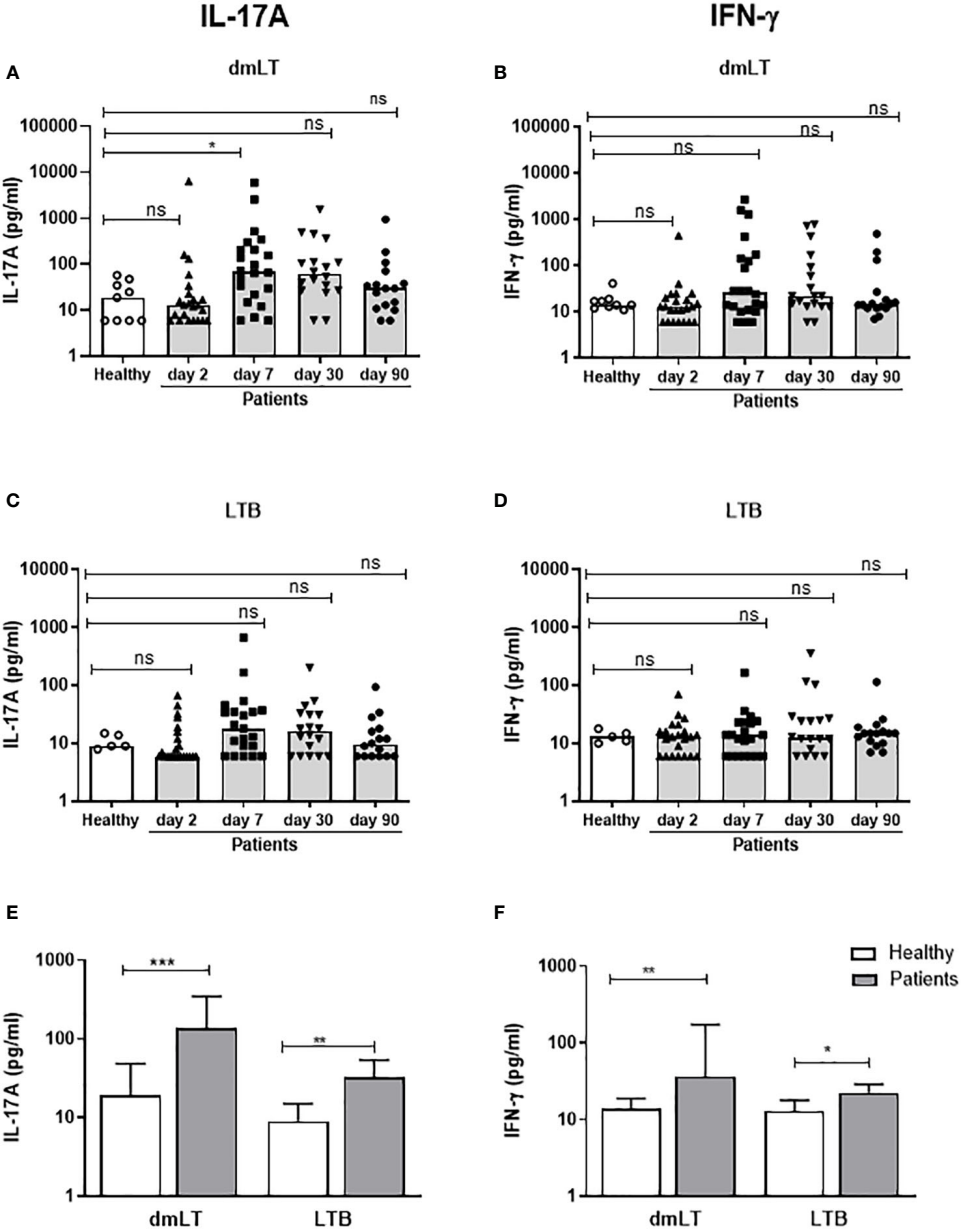
IL-17A and IFN-γ responses to dmLT and LTB in PBMCs from ETEC infected patients and healthy adults. IL-17A **(A, C, E)** and IFN-γ **(B, D, F)** concentrations in cultures with PBMCs stimulated with dmLT **(A, B)** and LTB **(C, D)** in healthy controls (n=5-9) and diarrheal patients on day 2, 7, 30 and 90 after hospitalization **(A, B)**; n=16-23 and **(C, D)**; n=16-22. **(A-D)** Responses at each individual time point. Each symbol represents one individual, and bars indicate median values. **(E, F)** Maximum responses at any time point. Bars represent median + 95% confidence interval. Statistical analysis was performed using the Kruskal Wallis test with Dunn’s multiple comparison post test **(A-D)** or Mann-Whitney test **(E**, **F)**. ^*^ *P <*0.05, ^**^ *P <*0.01, ^***^ *P <*0.001, ns; not significant; *P >*0.05.

T cell responses to LT were seen more clearly after stimulation with dmLT than LTB. A few patients with LT+ ETEC infection had IL-17A responses against dmLT or LTB on day 2 (9-13%), but significant responses to dmLT were observed first on day 7 after hospitalization, when 41% of the participants showed elevated IL-17A levels compared to controls (**Figure 4A** and **Table 3**). Responses peaked on day 7, but remained elevated until day 30 in 22% of patients (P>0.05 compared to day 2). In most patients, IL-17A responses returned to the levels observed in healthy controls on day 90. A trend for increased IL-17A responses after stimulation with LTB was also observed on day 7 (**Figure 4C**) when 33% patients responded in comparison to healthy controls (**Table 3**). When the maximum levels of IL-17A responses at any time point of infection were analyzed, increased IL-17A responses were found against both dmLT (56%) and LTB (45%) (**Figure 4E** and **Table 3**). Depletion of CD4+ Th cells from PBMCs isolated on day 7 after infection resulted in almost complete loss of IL-17A production in response to stimulation with dmLT and LTB (**Supplementary Figures 2A, B**), supporting that the IL-17A responses to LT were derived from CD4+ Th cells.

**Table 3:** Frequencies of IL-17A and IFN-γ responders^a^ against dmLT, LTB and CF antigens in ETEC infected patients.

	Day 2	Day 7	P-value^b^	Day 30	P-value^b^	Day 90	P-value^b^	Cumulative (Day 2/7/30/90)
IL-17A								
**dmLT**	3/23 (13%)	9/22 (41%)	0.047	4/18 (22%)	0.679	2/16 (13%)	1.000	13/23 (56%)
**LTB**	2/22 (9%)	7/21 (33%)	0.069	3/18 (17%)	0.642	1/16 (6%)	1.000	10/22 (45%)
**CS6**	–	3/10 (30%)	–	2/9 (22%)	–	1/7 (14%)	–	5/10 (50%)
**CS5**	–	3/7 (43%)	–	0/6 (0%)	–	0/4 (0%)	–	3/7 (43%)
**CFA/I**	–	0/5 (0%)	–	1/3 (33%)	–	0/4 (0%)	–	1/5 (20%)
IFN-γ								
**dmLT**	1/23 (4%)	8/22 (36%)	0.010	5/18 (28%)	0.070	3/16 (19%)	0.286	10/23 (43%)
**LTB**	1/22 (5%)	1/21 (5%)	1.000	3/18 (17%)	0.310	1/16 (6%)	1.000	4/22 (18%)
**CS6**	–	0/10 (0%)	–	0/9 (0%)	–	0/7 (0%)	–	0/10 (0%)
**CS5**	–	0/7 (0%)	–	0/6 (0%)	–	0/4 (0%)	–	0/7 (0%)
**CFA/I**	–	0/5 (0%)	–	1/3 (33%)	–	0/4 (0%)	–	1/5 (20%)

a A responder was defined as having ≥2-fold higher cytokine response compared to the mean+2SD cytokine concentration of the group of healthy controls.

b Statistical analysis was performed using the Fisher’s Exact test to compare the responder frequency between day 7, 30 and 90 vs day 2, when day 2 samples were available (stimulation with dmLT and LTB).

We also analyzed IFN-γ responses to dmLT and LTB in patients and controls. Neither dmLT- nor LTB-stimulation induced significant magnitudes of IFN-γ responses at any individual time point after infection (**Figures 4B, D**), but the responder frequencies to dmLT were significantly higher on day 7 compared to day 2 (36 vs 4%, **Table 3**). When maximum magnitudes of responses were evaluated at any time point, patients had significantly higher IFN-γ responses against both dmLT and LTB compared to healthy individuals (**Figure 4F**), although the median magnitudes of responses were lower than for IL-17A. Maximum increases in magnitudes (fold-rises) of IL-17A responses to dmLT or LTB did not correlate significantly with increases in magnitudes of IFN-γ responses to the same antigens (**Supplementary Figure 3**).

We have previously shown that dmLT enhances IL-17A responses to both antigens and mitogens/superantigens *in vitro* (29–31). To determine if the strong IL-17A response observed after stimulation with dmLT compared to LTB was related to the adjuvant effect of dmLT, cells from patients infected by LT+ ETEC were also stimulated with native LT and the LT-derivative LTE112K, which has a single mutation in the A-subunit that inhibits the ability of the molecule to induce cAMP and act as an oral adjuvant (38, 39). First, the adjuvant activity of dmLT and LTE112K was evaluated by stimulating PBMCs with the mitogen PHA with or without addition of dmLT or LTE112K. Only dmLT enhanced IL-17A responses to PHA (**Supplementary Figure 4A**), confirming that dmLT but not LTE112E has adjuvant function. We did not measure IFN-γ responses, since our previous studies have shown that dmLT cannot enhance IFN-γ responses *in vitro* (29–31). Next, the responses to dmLT on day 2 and day 7 were compared to responses induced by native LT or the other homologues LTE112K and LTB. dmLT, LT and LTE112K all induced significantly increased levels of IL-17A on day 7 compared to day 2 (**Supplementary Figure 4B**, P=0.02, P=0.01, P=0.02, respectively). IFN-γ responses were weaker and more variable between patients and only significant for dmLT and LT (P=0.01, P=0.03, respectively; **Supplementary Figure 4C**). Magnitudes of IL-17A and IFN-γ responses to LT and LTE112K were comparable to the responses induced by dmLT, whereas responses to LTB were significantly lower (**Supplementary Figures 4B, C**). Responses to LTB produced in the same laboratory (denoted by LTB2) as LT and LTE112K were also comparable to responses induced by stimulation with the LTB used in all other T cell and antibody analyses in the study (denoted by LTB1). The similar cytokine responses to dmLT and LTE112K, but lower responses to LTB, indicate that the strong response to dmLT is not directly related to the enzymatic effect of the A subunit included to the dmLT molecule.

Taken together, these results suggest that ETEC infected patients mount Th responses to LT that are predominantly of the Th17 type and can be detected at maximal levels 7 days after hospitalization.

### 3.4 CF-specific Th responses

**Figure 5:**
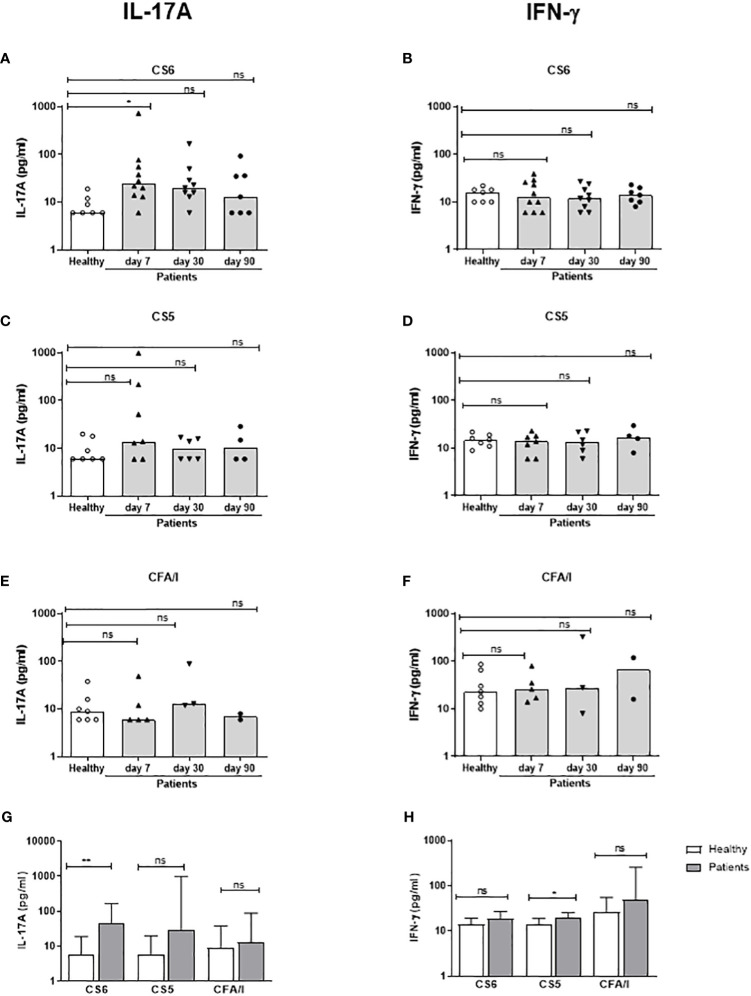
IL-17A and IFN-γ responses to CFs in PBMCs from ETEC infected patients and healthy adults. IL-17A **(A, C, E, G)** and IFN-γ **(B, D, F, H)** concentrations in cultures with PBMCs stimulated with CS6 **(A, B)**, CS5 **(C, D)** and CFA/I **(E, F)** in healthy controls (n=7) and diarrheal patients **(A, B)**; n=7-10, **(C, D)**; n=4-7, **(E, F)**; n=2-5) on day 7, 30 and 90 after hospitalization. **(A-F)** Responses at each individual time point. Each symbol represents one individual, and bars indicate median values. **(G, H)** Maximum responses at any time point. Bars represent median + 95% confidence interval. Statistical analysis was performed using the Kruskal Wallis test with Dunn’s multiple comparison post test **(A-F)** or the Mann-Whitney test **(G, H)**. ^*^ *P <*0.05, ^**^ *P <*0.01, ns, not significant; *P >*0.05.

We also analyzed Th responses to CFs expressed by the infecting strains on day 7, 30 and 90 after hospitalization. We could not measure CF-specific T cell responses on day 2, since the CF expression of the infecting ETEC strain was not known at this time point and the cell numbers did not allow stimulation with several different CFs. Patients infected with CS6+ ETEC had significantly increased magnitudes of IL-17A responses against CS6 on day 7 compared to healthy controls (30% responder frequency) but responses remained elevated in only a few patients on day 30-90 (**Figure 5A** and **Table 3**). Some patients responded with increased levels IL-17A production to stimulation with CS5 and CFA/I on day 7, however, the number of patients tested for these antigens was small (n=3-7) and responses were not significant (**Figures 5C, E**). Considering the maximum IL-17A responses at any time point, increased responses were again only observed against CS6 (**Figure 5G**). Depletion of CD4+ Th cells from PBMCs isolated on day 7 after infection substantially reduced IL-17A production in response to stimulation with CS6 and CS5 (**Supplementary Figure 2C**), supporting that the observed IL-17A responses to CFs were derived from CD4+ Th cells. Patients had almost no detectable CF-specific IFN-γ responses at any time point after infection (**Figures 5B, D, F**). However, a very small but significant increase in IFN-γ was found for CS5 when maximum responses at any time point were considered (**Figure 5H**).

### 3.5 Correlations between IgA and cytokine responses

**Figure 6:**
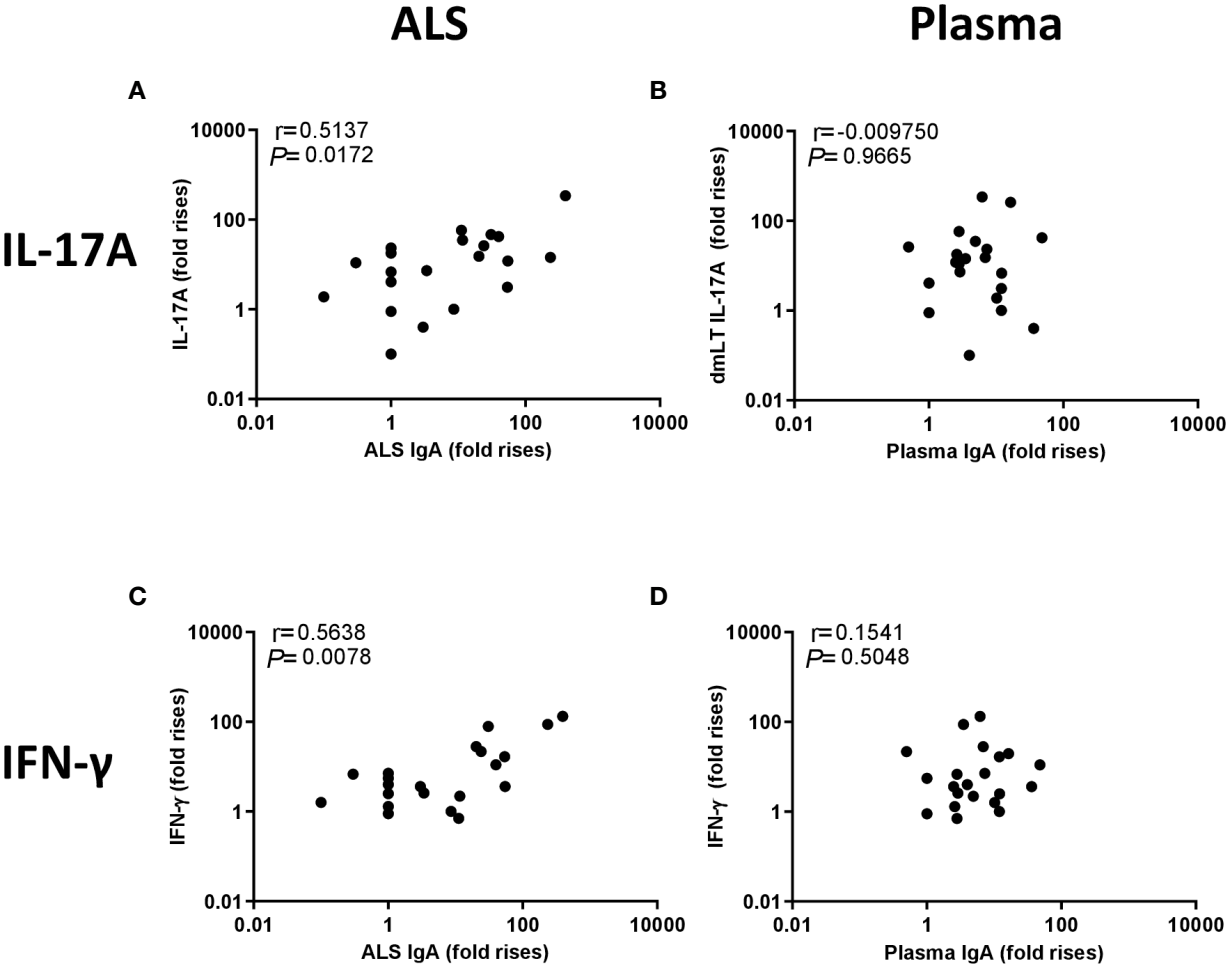
Correlation between IL-17A and IFN-γ responses to dmLT and IgA responses to LTB in ALS and plasma. Correlation between the fold-increases in cytokines and IgA levels (maximum responses at any time point divided by the response on day 2). **(A)** IL-17A response to dmLT vs. ALS IgA to LTB, **(B)** IL-17A response to dmLT vs plasma IgA to LTB, **(C)** IFN-γ response to dmLT vs. ALS IgA to LTB and **(D)** IFN-γ response to dmLT vs. plasma IgA to LTB. Statistical analysis was performed using the Spearman test.

We next evaluated the relationship between the IgA and T cell cytokine responses induced by ETEC infection. We first investigated the correlation between the maximum increases in magnitudes (fold-rises between maximum responses at any time point of infection versus day 2) of LTB-specific ALS and plasma IgA responses versus the maximum increases in magnitudes of IL-17A and IFN-γ responses induced by stimulation with dmLT, resulting in several significant correlations, as described below. Correlation analyses were also performed using the maximal response level minus the level measured on day 2, but these analyses did not reveal any significant correlations (data not shown). The magnitudes (fold-rises) of ALS IgA responses to LTB correlated significantly with the IL-17A responses (r=0.51, P=0.02) (**Figure 6A**) as well as IFN-γ responses (r=0.56, p=0.01) (**Figure 6C**) to dmLT (**Figure 6C**). In contrast, plasma IgA LTB antibody responses did not correlate with either the IL-17A or IFN-γ responses to dmLT (**Figures 6B, D**). We also analyzed the correlation between the weaker IL-17A and IFN-γ responses to LTB versus ALS and plasma IgA antibody responses to the same antigen. Significant correlations were observed between ALS IgA and IFN-γ responses to LTB (r=0.50, p=0.02) (**Supplementary Figure 5**). These results support a relation between both Th17 and Th1 responses and ASC-IgA responses, most likely reflecting mucosal IgA responses to ETEC.

## 4 Discussion

Although T cells are likely to play an active role in promoting the development of SIgA-mediated protection against ETEC, T cell responses against natural ETEC infection have, to our knowledge, not previously been investigated. In this study, we analyzed Th responses in hospitalized Bangladeshi patients with ETEC diarrhea during acute infection and convalescence. Consistent with previous studies, the patients showed significant IgA ASC as well as plasma IgA responses to LTB and CFs expressed by the infecting strains (9–11). The IgA responses were concomitant with significantly higher frequencies of circulating Th cells expressing chemokine receptors associated with a Th17 cytokine profile (CCR6+CXCR3-) in blood during the early phase of ETEC infection and convalescence (day 2 and 7), whereas the proportions of cells with Th1 or Th2 phenotypes did not change. The induction of ETEC-specific Th17 responses was further supported by the increases in IL-17A secretion from PBMCs in response to stimulation with dmLT, LT, LTB and CS6 in ETEC patients expressing the corresponding antigens. We also observed IL-17A responses to CS5 and CFA/I in some patients, but due to lower prevalence of ETEC strains expressing these CFs in the study group, smaller numbers of participants were tested for responses to these antigens and larger studies are needed to fully characterize T cell responses to CS5 and CFA/I.

Th17 cells represent the most abundant cellular source of IL-17A in human gut tissue (20), but IL-17A can also be produced from innate cells (40). Results from depletion experiments verified that the IL-17A detected in our experiments was mainly produced by CD4+ T cells. IFN-γ responses to ETEC antigen stimulation were lower in magnitude and less frequent compared to IL-17A responses. IFN-γ is mainly produced by activated T cells, particularly Th1 cells as well as innate cells like natural killer cells, macrophages and dendritic cells (41). Due to low and infrequent IFN-γ responses observed in ETEC infected patients, we were not able to determine the cellular source of IFN-γ in our study.

Our results are consistent with a few previous reports of T cell responses in experimental ETEC human challenge models. After challenge of Norwegian volunteers with the ETEC strain TW10722, which expresses ST, CS5 and CS6, but not LT, Th responses to CS5 and CS6 were detected in peripheral blood, with particularly strong and long-lived responses to CS5 (42). Challenge with the TW11681 strain, that expresses ST, CFA/I and CS21, also induced responses to CFA/I in some individuals (43). In both studies, T cell responses to the *E. coli* mucinase YghJ expressed by both strains were also noted (42, 43). However, in these studies the Th responses were analyzed using a flow cytometric assay based on expression of Th activation markers after antigen stimulation, and the cytokine profiles of the ETEC specific cells were not determined. A very small flow cytometric study of CD4+ T cell responses to the LT+ ST+CFAI/+ H10407 ETEC challenge strain in American volunteers supported the presence of Th cells specific for CFA/I in peripheral blood of ETEC infected individuals with mild diarrhea, whereas such responses were not observed in patients with more severe symptoms (44). The ETEC-specific cells predominantly produced TNF-α and IL-2, but IFN-γ and IL-17A responses were also detected in some individuals. However, since only five participants were included in the T cell analysis, it is difficult to draw any firm conclusions of the general characteristics or the potentially protective effect of T cells from these results. Experimental infection with the same ETEC H10407 strain has also been shown to induce significantly increased levels of IL-17A and IFN-γ in serum of volunteers who developed symptomatic infection (45). The cellular source of the serum cytokines was not defined and since the serum cytokine levels increased very early after challenge, innate cells may be involved. However, early IL-17A production has also been described in mucosal CD3+CD4+ T cells after infection of naïve piglets with ETEC expressing F4 fimbriae (46). The animals also mounted ETEC specific IL-17A and IFN-γ responses in peripheral blood, as revealed by analysis of cytokine mRNA in unstimulated PBMCs from infected animals as well as by analysis of cytokines in culture supernatants from antigen stimulated PBMCs. ETEC is an important diarrheal pathogen in pigs, and although the ETEC strains which infect pigs do not infect humans, several studies confirm similar pathogenic mechanisms of ETEC in pigs and humans and also support the importance of SIgA responses in protection against the infection (47, 48). Collectively, our studies of T cell responses to natural ETEC infection, and the results from different human and animal ETEC challenge models, support that ETEC induce Th responses to both LT and CFs. Furthermore, Th17 responses seem to be an important component of the response both in naïve people and individuals living in an ETEC endemic area, who are most likely are primed by previous ETEC infections.

It was noteworthy that the IL-17A responses in ETEC patients infected by LT positive strains in our study were stronger after stimulation with dmLT and LT compared to LTB. LT and dmLT can act both as antigens and adjuvants and can boost both T and B cell responses to various types of vaccines, including ETVAX, in animals as well as humans (7, 25, 28). We have also previously shown that LT, dmLT and cholera toxin (CT) primarily promote Th17 but not Th1 type responses to both vaccine antigens and polyclonal stimuli *in vitro*, whereas LTB does not have any adjuvant effect (29–31). Since we found that stimulation with the LT mutant LTE112K, which contains an A subunit which lacks ability to induce cAMP due to a single amino acid substitution and has no adjuvant activity when administered orally (38, 39), gave rise to similar strong IL-17A responses as dmLT or LT, our results indicate that the strong T cell response to dmLT and LT compared to LTB may not be directly related to the adjuvant effect. The stronger T cell responses to dmLT may instead be a result of responses to T cell epitopes in the A subunit, and/or be related to increased binding, uptake and presentation of dmLT by antigen presenting cells. Antibodies to the A subunit have been demonstrated in ETEC infected individuals (49), and the A subunit has also been shown to have adjuvant function independently of the B subunits (50). These observations also indicate that the inclusion of dmLT in the ETVAX vaccine formulation may be an advantage not only from the perspective of its adjuvant potential, but also given it ability to induce responses to the A subunit of the LT toxin, which may be an important contributor to vaccine induced protection. To gain further mechanistic insights into how protective immunity to ETEC is induced, T- and B cell responses to both the A- and B-subunits of LT should be monitored in future studies.

ETEC LT has 80% structural similarity to CT produced by *Vibrio cholerae* O1 and causes diarrhea by similar mechanisms. The SIgA antibodies against both CT and somatic cholera antigens such as lipopolysaccharide (LPS) and toxin-coregulated pilus (TCP) are thought to contribute to protection against cholera (51). Interestingly, natural cholera infection has been shown to induce IL-17A responses in duodenal tissue from adult Bangladeshis early after hospitalization (day 2) and the responses remained until day 30 (52). A marked increase in IL-17A and IFN-γ production was also noted in whole blood cultures from cholera patients after stimulation with a membrane protein preparation (MP) of cholera antigens (52). In Bangladeshi children, MP as well as CT, CT B subunit (CTB), dmLT and a single mutant CT molecule (mCT) with retained adjuvanticity, but less binding to GM1, have been shown to induce significant Th cell proliferation, and mCT-stimulation of cells in whole blood cultures was also demonstrated to result in significant IL-17A, IFN-γ and IL-13 responses (16). These results suggest that ETEC and cholera both induce T cell responses of the Th17 type, but also indicate that Th1 responses may be stronger after cholera compared to ETEC infection.

Th17 cells have been implicated in mediating protective immunity against a variety of additional bacterial infections in the intestinal tract, including *Salmonella enterica* serovar Typhimurium, *Helicobacter pylori* and *Citrobacter rodentium* (19, 53–55). Th17 cells may contribute to protection by different mechanisms, including induction of inflammation via recruitment of neutrophils, enhancement of production of antimicrobial peptides or by promotion of the development of IgA responses or secretion of IgA over the mucosal epithelium, and the importance of these mechanisms are likely to vary considerably between different infections (20, 56). We demonstrate here that the magnitudes of both IL-17A and IFN-γ responses to dmLT and IFN-γ responses to LTB correlate with the magnitude of ASC responses to LTB measured by the ALS method. Although the correlations were moderate r=(0.51-0.56), these results indicate that both types of Th responses may be involved in promoting development of B cell responses. In contrast, no correlation was seen between T cell and plasma IgA responses. The proportions of patients responding to dmLT with IL-17A responses and IgA LTB were comparable (about 50%), whereas ALS IgA responses to CS6 and CS5 were more frequent than T cell responses to the same antigens (80-90% compared to 40-50%). However, it is possible that differences in kinetics between T and B cell responses may have influenced these results. Although maximal cytokine as well as ALS responses were detected on day 7 in our study, the enrolled patients had experienced ETEC symptoms 6-96 h (median 24 hours) before being hospitalized and sampled, and we may have missed transient responses or analyzed submaximal responses in some individuals.

Vaccines against ETEC currently under development are aiming to induce mucosal SIgA responses against both CFs and toxins. ETEC bacteria are non-invasive and localized in the gut, therefore mucosal SIgA antibodies directed against CFs and LTB are considered to confer immune protection after natural infection or vaccination (2, 6). In a number of different studies we have shown that the oral ETEC vaccine ETVAX induces both blood ASC responses, characterized by prominent expression of integrin α4β7, and fecal SIgA against LT, CFs, and LPS and that such responses are promoted by addition of dmLT in both mice and humans (6, 7, 23, 25, 57). We have also shown that the vaccine induces both Th1 and Th17 responses to LTB and CFs, and that the strongest CS6 specific T cell as well as ASC responses were detected in those participants receiving vaccine with dmLT (25) and Lundgren A, unpublished). The results from the current study support the similarity between both T and B cell responses induced by ETVAX and natural ETEC infection, indicating that the vaccine is able to induce protective responses. In addition, we have previously shown in Swedish vaccinees that activated Tfh-like cells are released in peripheral blood after ETVAX vaccination and that the magnitude of the response correlated with development of plasmablast responses and long-lived B cell memory (23). Tfh cells are located in the germinal centers, where they promote isotype switching, affinity maturation and development of long lived plasma cells and memory B cells. Circulating Tfh-like (cTfh) cells clonally related to Tfh cells in lymph nodes have been demonstrated and have been shown to be associated with development of highly functional antibodies to both vaccination and infection (58–60). cTfh cells were not evaluated specifically in the present study, but since cTfh have been shown to be able to produce both IL-17A and IFN-γ (61), some of the cytokines detected here may be produced by cTfh cells. Increased proportions of ETEC-specific cTfh cells have been observed in participants challenged with ST+LT+ ETEC (44). cTfh responses to natural ETEC infection will be specifically investigated in our continued studies of immunity to ETEC infection.

In conclusion, we have shown that natural ETEC infection in adults living in an ETEC endemic area gives rise to Th responses to both LT and CFs. Responses appear to be dominated by Th17 cells, but magnitudes of both IL-17A and IFN-γ responses correlate with ASC IgA responses, most likely derived from and homing to intestinal mucosa, supporting that several Th subtypes may be important in the development of intestinal IgA responses against ETEC. Our study has several strengths, particularly the longitudinal analysis of antigen specific Th cells in the same individuals at several time points during both acute infection and convalescence, the comprehensive comparison of Th responses with both circulating ASC and plasma IgA responses, and the ETEC endemic study setting, which enabled analysis of immune responses to natural ETEC infection in previously ETEC primed subjects. Study limitations include the low number of patients infected with CFA/I and CS5 expressing ETEC strains enrolled in the study and that most patients had severe dehydration, which limits the possibility to investigate the relation of disease severity and immune responses. Methodological limitations include that mucosal IgA responses were estimated by measurement of antigen specific ASCs migrating in peripheral blood rather than by direct evaluation of SIgA responses in fecal samples or mucosal biopsies, and that T cell responses were measured by analysis of cytokines in culture supernatants and not by flow cytometric analysis of intracellular cytokines, which may have provided additional information about the frequency and phenotype of antigen specific T cells. Further detailed characterization of T and B cell responses to ETEC infection in larger studies, including different age groups and populations with different levels of priming by natural ETEC infection, is likely to provide increased mechanistic insights into how protective immunity against ETEC develops and may also result in the identification of biomarkers useful for identifying the most promising vaccine candidates.

## Data availability statement

The raw data supporting the conclusions of this article will be made available by the authors, without undue reservation.

## Ethics statement

The studies involving humans were approved by Research review committee and Ethical review committee, icddr,b. The studies were conducted in accordance with the local legislation and institutional requirements. The participants provided their written informed consent to participate in this study.

## Author contributions

AL, MA, TB and FQ designed and planned the studies. MA, SB, NN and LH performed the immunological analyses. MA and AL wrote the manuscript. All authors contributed to the interpretation of results and critical review and revision of the manuscript and have approved the final version.

## Supplementary material

Supplementary Table 1 Frequencies of IgA antibody responders against LTB and CF antigens in ALS and plasma specimens from ETEC patients (comparison with day 2 responses^a^). ^a^A responder was defined as having ≥2-fold higher antibody titer on day 2/30/90 compared to day 2.

Supplementary Table 2 Frequencies of IL-17A and IFN-γ responders against LTB and dmLT in ETEC infected patients (comparison with day 2 responses^a^). ^a^A responder was defined as having ≥2-fold higher cytokine response on day 2/30/90 compared to day 2.
