# Supplementary material for: T helper cell responses in adult diarrheal patients following natural infection with enterotoxigenic Escherichia coli are primarily of the Th17 type
Source: Front Immunol. 2023 Sep 20;14:1220130. doi: 10.3389/fimmu.2023.1220130 (PMC10552643; doi:10.3389/fimmu.2023.1220130)
Supplement: Supplementary Table 1 — Frequencies of IgA antibody responders against LTB and CF antigens in ALS and plasma specimens from ETEC patients (comparison with day 2 responsesa). aA responder was defined as having ≥2-fold higher antibody titer on day 2/30/90 compared to day 2. [file Table_1.docx]

**Supplementary Table 1.** Frequencies of IgA antibody responders against LTB and CF antigens in ALS and plasma specimens from ETEC patients (comparison with day 2 responses^a^).

|  | **Day 7** | **Day 30** | **Day 90** | **Cumulative**  (**Day 7/30/90)** |
| --- | --- | --- | --- | --- |
| **ALS** | | | | |
| LTB | 14/25 (56%) | 1/20 (5%) | - | 14/26 (54%) |
| CS6 | 11/12 (92%) | 1/10 (10%) | - | 11/12 (92%) |
| CS5 | 9/11 (82%) | 2/9 (22%) | - | 9/11 (82%) |
| CFA/I | 5/6 (83%) | 2/5 (40%) | - | 5/6 (83%) |
| **Plasma** | | | | |
| LTB | 20/25 (80%) | 11/22 (50%) | 3/19 (16%) | 22/26 (85%) |
| CS6 | 20/25 (80%) | 11/22 (50%) | 3/19 (16%) | 22/26 (85%) |
| CS5 | 10/11 (91%) | 5/9 (56%) | 3/7 (43%) | 10/11 (91%) |
| CFA/I | 5/6 (83%) | 3/5 (60%) | 1/3 (33%) | 5/6 (83%) |

^a^A responder was defined as having ≥2-fold higher antibody titer on day 7/30/90 compared to day 2.
