# Supplementary material for: T helper cell responses in adult diarrheal patients following natural infection with enterotoxigenic Escherichia coli are primarily of the Th17 type
Source: Front Immunol. 2023 Sep 20;14:1220130. doi: 10.3389/fimmu.2023.1220130 (PMC10552643; doi:10.3389/fimmu.2023.1220130)
Supplement: Supplementary Table 2 — Frequencies of IL-17A and IFN-γ responders against LTB and dmLT in ETEC infected patients (comparison with day 2 responsesa). aA responder was defined as having ≥2-fold higher cytokine response on day 2/30/90 compared to day 2. [file Table_2.docx]

**Supplementary Table 2.** Frequencies of IL-17A and IFN-γ responders against LTB and dmLT in ETEC infected patients (comparison with day 2 responses^a^).

|  | **Day 7** | **Day 30** | **Day 90** | **Cumulative**  (**Day 7/30/90)** |
| --- | --- | --- | --- | --- |
| **IL-17A** | | | | |
| dmLT | 12/22 (55%) | 13/18 (72%) | 9/16 (56%) | 16/23 (70%) |
| LTB | 8/21 (38%) | 10/18 (56%) | 3/16 (19%) | 15/22 (68%) |
| **IFN-γ** | | | | |
| dmLT | 12/22 (55%) | 8/18 (44%) | 8/16 (50%) | 16/23 (70%) |
| LTB | 3/21 (14%) | 6/18 (33%) | 6/16 (38%) | 9/22 (41%) |

^a^A responder was defined as having ≥2-fold higher cytokine response on day 7/30/90 compared to day 2.
