# Supplementary material for: T helper cell responses in adult diarrheal patients following natural infection with enterotoxigenic Escherichia coli are primarily of the Th17 type
Source: Front Immunol. 2023 Sep 20;14:1220130. doi: 10.3389/fimmu.2023.1220130 (PMC10552643; doi:10.3389/fimmu.2023.1220130)
Supplement: Supplementary file 3 [file Presentation_1.pptx]

## Slide 1
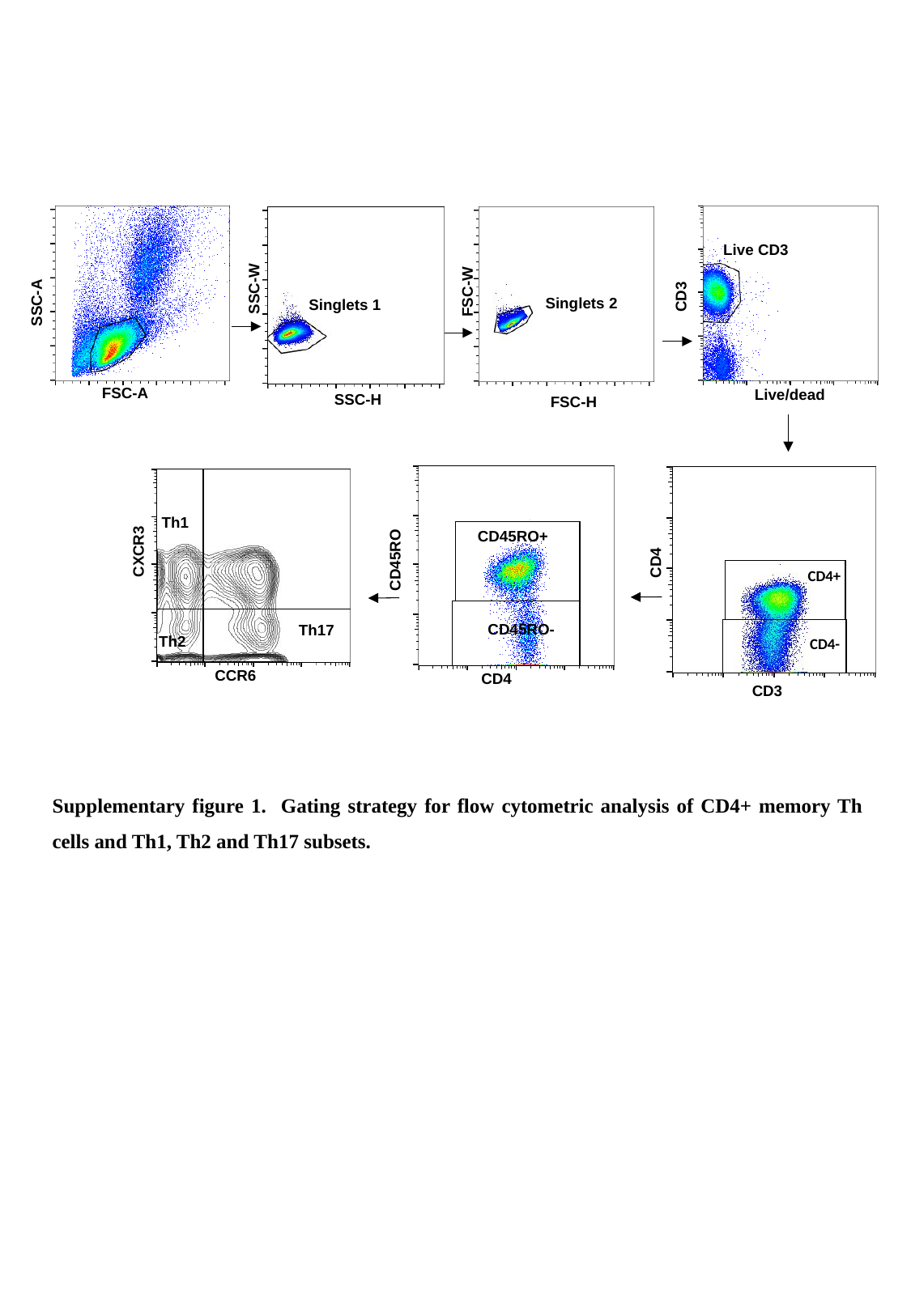

CD3
Live/dead
Live CD3
SSC-W
SSC-H
FSC-W
SSC-A
Singlets 2
Singlets 1
FSC-A
FSC-H
CD45RO
CD4
CD4+
CD4-
CD3
Th1
CXCR3
Th17
Th2
CCR6
CD45RO+
CD45RO-
CD4
Supplementary figure 1. Gating strategy for flow cytometric analysis of CD4+ memory Th cells and Th1, Th2 and Th17 subsets.

## Slide 2
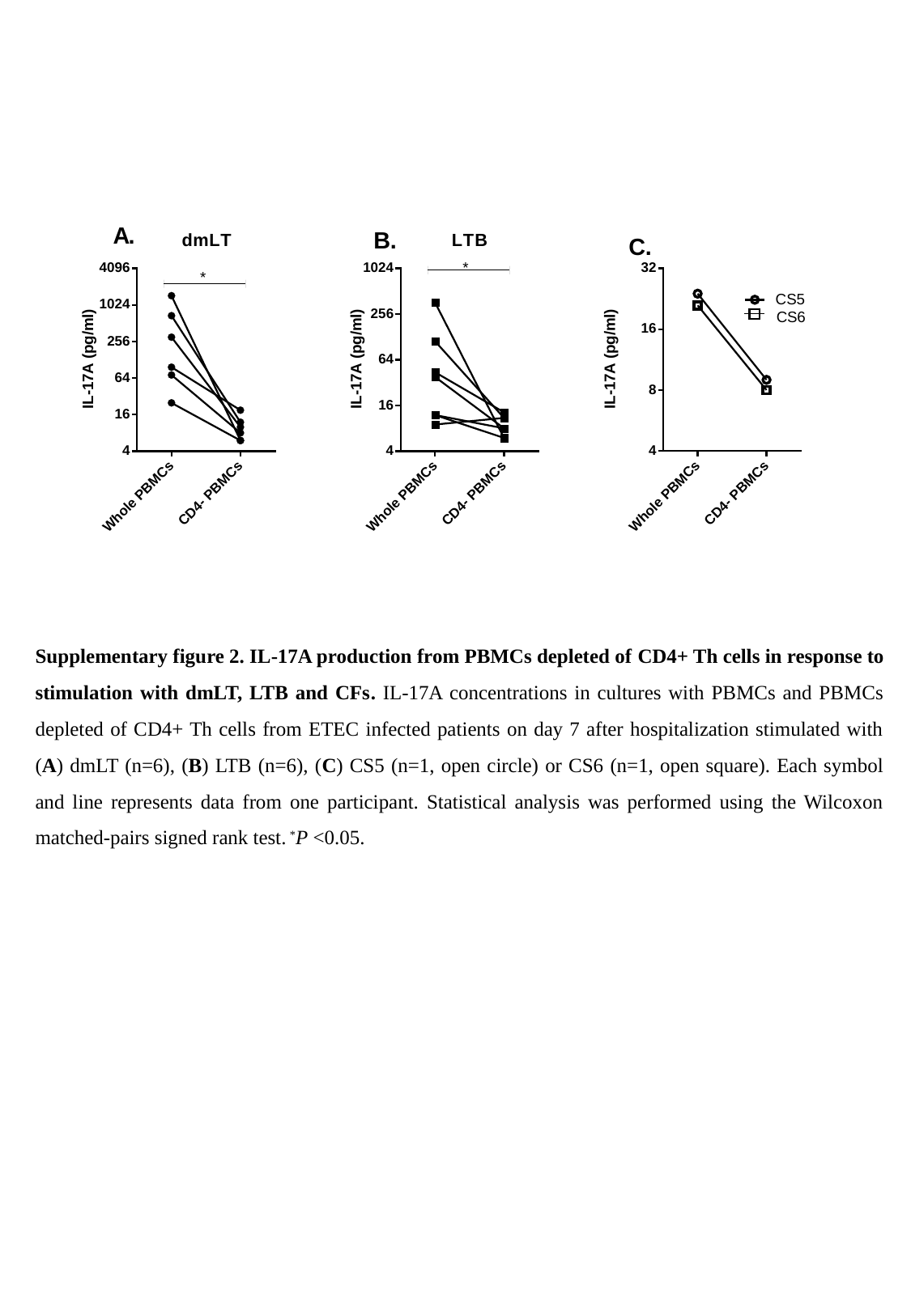

Supplementary figure 2. IL-17A production from PBMCs depleted of CD4+ Th cells in response to stimulation with dmLT, LTB and CFs. IL-17A concentrations in cultures with PBMCs and PBMCs depleted of CD4+ Th cells from ETEC infected patients on day 7 after hospitalization stimulated with (A) dmLT (n=6), (B) LTB (n=6), (C) CS5 (n=1, open circle) or CS6 (n=1, open square). Each symbol and line represents data from one participant. Statistical analysis was performed using the Wilcoxon matched-pairs signed rank test. *P <0.05.

## Slide 3
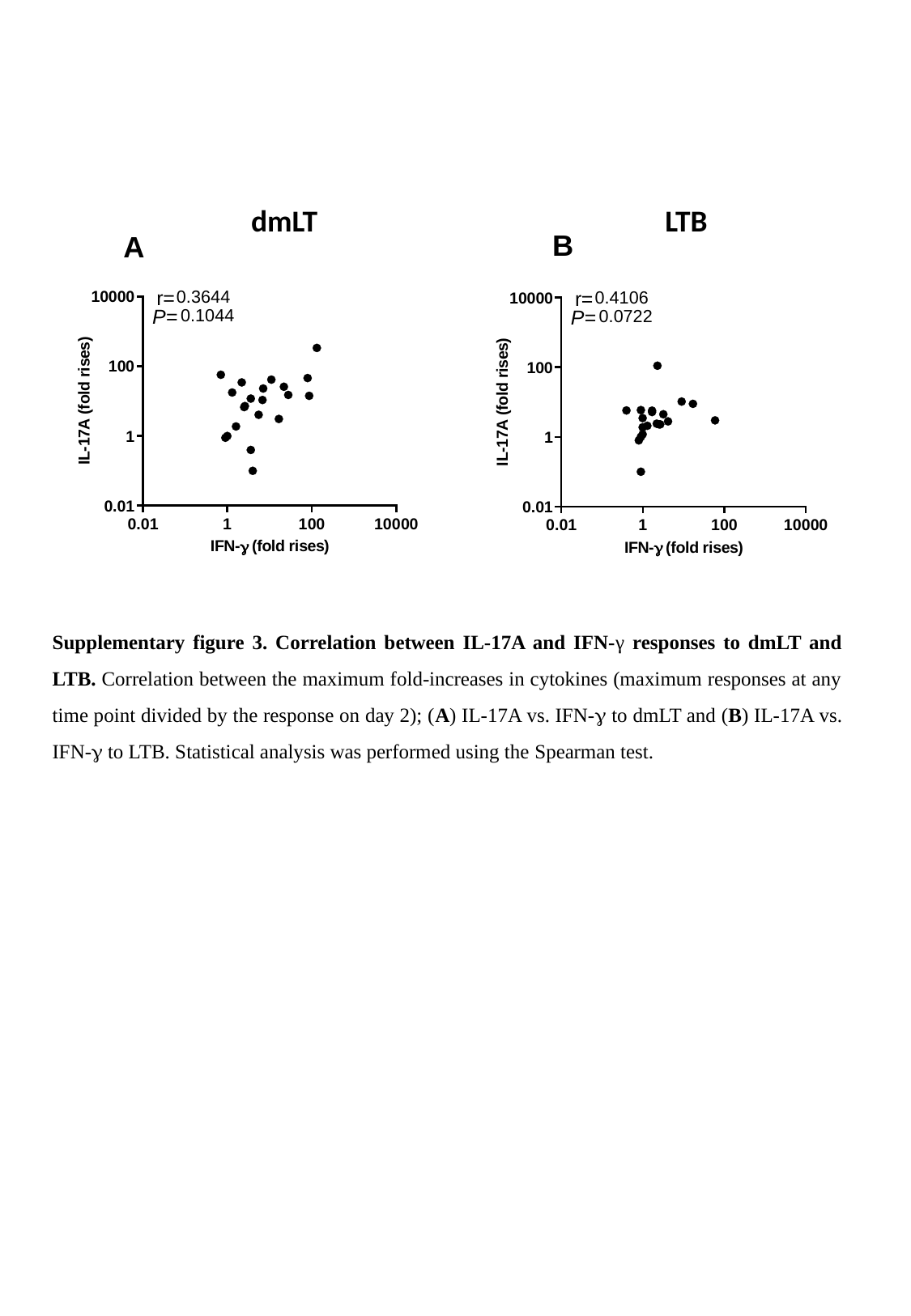

dmLT
LTB
Supplementary figure 3. Correlation between IL-17A and IFN-γ responses to dmLT and LTB. Correlation between the maximum fold-increases in cytokines (maximum responses at any time point divided by the response on day 2); (A) IL-17A vs. IFN-γ to dmLT and (B) IL-17A vs. IFN-γ to LTB. Statistical analysis was performed using the Spearman test.

## Slide 4
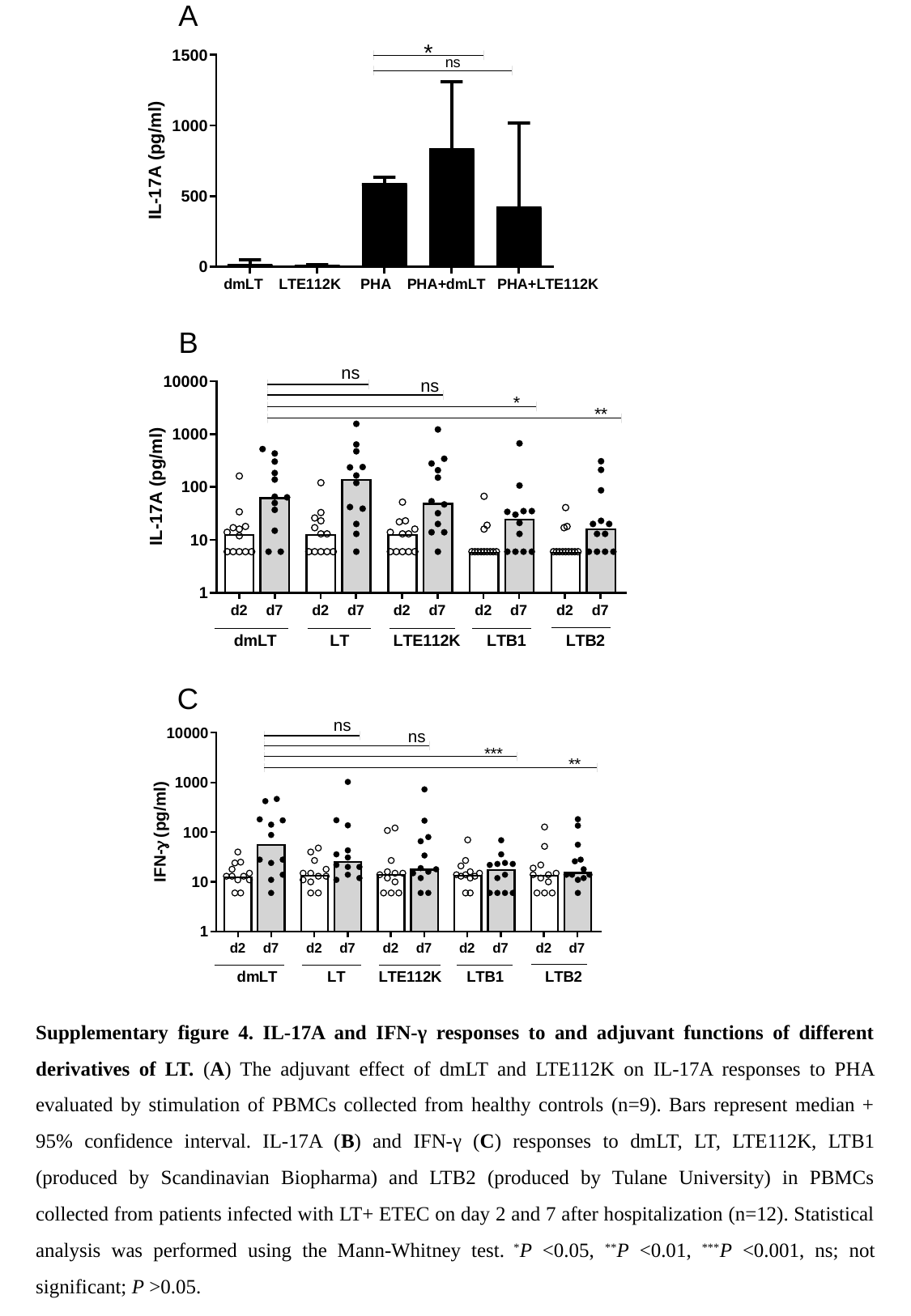

Supplementary figure 4. IL-17A and IFN-γ responses to and adjuvant functions of different derivatives of LT. (A) The adjuvant effect of dmLT and LTE112K on IL-17A responses to PHA evaluated by stimulation of PBMCs collected from healthy controls (n=9). Bars represent median + 95% confidence interval. IL-17A (B) and IFN-γ (C) responses to dmLT, LT, LTE112K, LTB1 (produced by Scandinavian Biopharma) and LTB2 (produced by Tulane University) in PBMCs collected from patients infected with LT+ ETEC on day 2 and 7 after hospitalization (n=12). Statistical analysis was performed using the Mann-Whitney test. *P <0.05, **P <0.01, ***P <0.001, ns; not significant; P >0.05.

## Slide 5
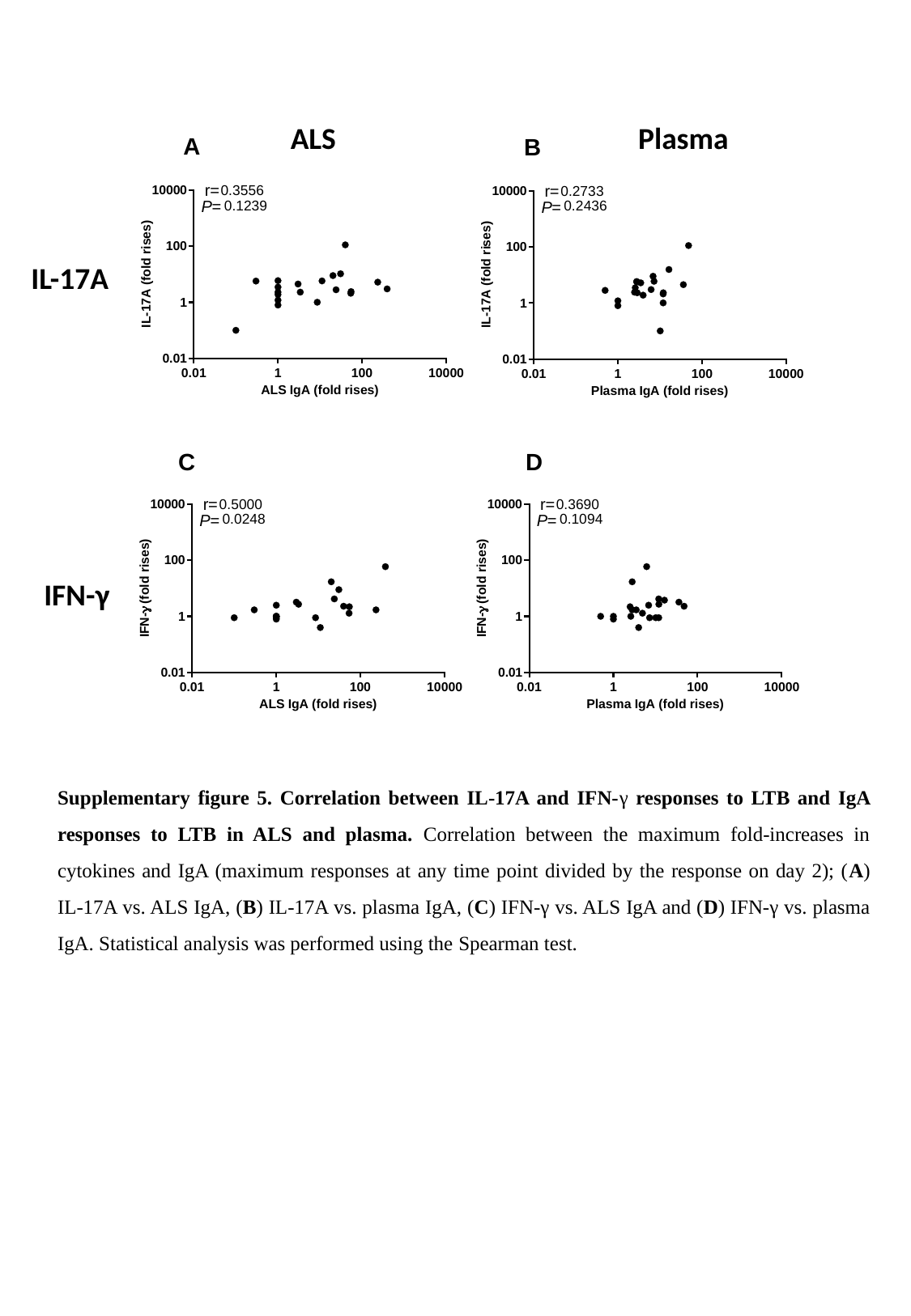

ALS
Plasma
IL-17A
IFN-γ
Supplementary figure 5. Correlation between IL-17A and IFN-γ responses to LTB and IgA responses to LTB in ALS and plasma. Correlation between the maximum fold-increases in cytokines and IgA (maximum responses at any time point divided by the response on day 2); (A) IL-17A vs. ALS IgA, (B) IL-17A vs. plasma IgA, (C) IFN-γ vs. ALS IgA and (D) IFN-γ vs. plasma IgA. Statistical analysis was performed using the Spearman test.
